# The use of transcranial direct current stimulation (tDCS) to reduce impulsivity and aggression in adults with mild intellectual developmental disabilities: the tDCS-RIADD randomised controlled trial protocol

**DOI:** 10.1186/s13063-022-06350-5

**Published:** 2022-05-23

**Authors:** Najat Khalifa, Emily R. Hawken, Andrew Bickle, Mariel Cabrera, Travis Heath, Andrew Drury, Jessica Jones, Muhammad Ayub

**Affiliations:** 1 Department of Psychiatry, Queen’s University, Kingston, Ontario Canada; 2 Providence Care Hospital, Kingston, Ontario Canada; 3 Department of Psychology, Queen’s University, Kingston, Ontario Canada; 4 University College London, London, UK

**Keywords:** tDCS, Aggression, Impulsivity, Developmental disability, Neurostimulation

## Abstract

**Background:**

Challenging behaviours, in particular aggressive behaviours, are prevalent among people with intellectual developmental disabilities. Predictors of challenging behaviours are numerous, including past history of aggression, poor coping skills and impulsivity. Factors like motor or rapid-response impulsivity (RRI) have neurobiological underpinnings that may be amenable to change via neuromodulation using non-invasive brain stimulation techniques like transcranial direct current stimulation (tDCS).

**Methods:**

This study aims to determine the efficacy of anodal tDCS in reducing RRI and incidents of aggression in people with intellectual developmental disabilities (IDD) in residential or hospital settings. Using a single blind, randomised, sham-controlled trial design, adults with IDD, with a history of impulsivity leading to aggression, will be randomised to receive either repetitive anodal or sham tDCS applied to the left dorsolateral prefrontal cortex. Outcome measures assessing impulsivity and aggression will be collected for up to 1 month following the last tDCS session.

**Discussion:**

The results of this study may pave the way for developing targeted interventions for impulsivity and aggressive behaviours in people with IDD.

## Administrative information

Note: the numbers in curly brackets in this protocol refer to SPIRIT checklist item numbers. The order of the items has been modified to group similar items (see https://can01.safelinks.protection.outlook.com/?url=http%3A%2F%2Fwww.equator-network.org%2Freporting-guidelines%2Fspirit-2727-statement-defining-standard-protocol-items-for-clinical-trials%2F&data=04%7C01%7Cnrk2%40queensu.ca%7C7a5979f36f7f43b5866608da0eff7c2b%7Cd61ecb3b38b142d582c4efb2838b925c%7C1%7C0%7C637838787710809005%7CUnknown%7CTWFpbGZsb3d8eyJWIjoiMC4wLjAwMDAiLCJQIjoiV2luMzIiLCJBTiI6Ik1haWwiLCJXVCI6Mn0%3D%7C3000&sdata=PhMQm49uQnZT6a8rgoUxl70CjCar1zaViVyyqTgQGmI%3D&reserved=0).

**Table:** 

Title {1}	The use of transcranial Direct Current Stimulation (tDCS) to reduce impulsivity and aggression in adults with mild intellectual developmental disabilities: The tDCS-RIADD randomized controlled trial protocol.
Trial registration {2a and 2b}.	ClinicalTrials.gov Registration number: NCT04732052.
Protocol version {3}	January 7, 2022; Version 5
Funding {4}	This study is funded by the Department of Psychiatry, Queen’s University, Kingston, Ontario, Canada.
Author details {5a}	Najat Khalifa,^1,2^ Emily R Hawken, ^1,3^Andrew Bickle, ^1,2^ Mariel Cabrera^1^, Travis Heath^2^, Andrew Drury^1^, Jessica Jones, ^1,3^ & Muhammad Ayub^1,4^ ^1^Department of Psychiatry, Queen’s University, Kingston, Ontario, Canada ^2^Providence Care Hospital, Kingston, Ontario, Canada. ^3^Department of Psychology, Queen’s University, Kingston, Ontario, Canada. ^4^University College London, London, United Kingdom.
Name and contact information for the trial sponsor {5b}	Muhammad Ayub, *MD, MRCPsych* Department of Psychiatry Queens University Kingston 191 Portsmouth Avenue Kingston ON Canada K7M 8A6 Email: ma84@queensu.ca
Role of sponsor {5c}	The study sponsor contributed to the study inception, protocol design and manuscript editing. The funder had no role in study design; collection, management, analysis, and interpretation of data; writing of the report; and the decision to submit the report for publication.

## Introduction

### Background and rationale {6a}

Challenging  behaviours are common among people with intellectual developmental disabilities (IDD), encompassing behaviours such as aggression, sexually inappropriate behaviours, self-injury, and criminality [1]. Research studies reported an overall prevalence rate of 10–15% for any type of challenging behaviour [2] and rates ranging from 6.1% in community settings to 40% in long-stay hospitals with significant adverse consequences, e.g. in terms of quality of life, legal consequences, breakdown of placements, and harm to others [3].

The aetiology of aggressive behaviours is multi-factorial, including physical complaints (e.g. pain, constipation, infection), behavioural phenotypes (e.g. Prader Willi syndrome), psychiatric disorders (e.g. psychosis, autism spectrum disorder), and psychosocial factors like trauma [3]. Predictors of inpatient aggression among people with IDD are numerous, including past history of aggression, poor coping skills, and impulsivity [4, 5]. Some factors, like impulsivity, have defined neurobiological underpinnings that are amenable to change through biopsychosocial interventions.

A multi-dimensional construct with cognitive, motor, and temporal dimensions [6, 7], impulsivity reflects a tendency to act without thinking through the consequences of one’s actions. Motor or rapid-response impulsivity (RRI) is a form of impulsivity that reflects failure to refrain from action initiation or to stop an ongoing or prepotent action [8]. RRI underpins several psychiatric disorders including borderline personality disorder, attention deficit hyperactivity disorder, and substance use disorders. Due to its association with criminality, poor concordance with treatment, and suicidality, impulsivity is regarded as an important consideration in risk assessment and management [6, 9–12]. Although numerous biopsychosocial approaches have been proposed to manage aggression and impulsivity in people with IDD [3, 13], the evidence base for their efficacy is limited, highlighting the need to develop specific or adjunctive interventions.

Transcranial direct current stimulation (tDCS) has been used to modulate impulsivity with some promising results [7, 14, 15], offering great potential as a treatment modality for impulsive and aggressive behaviours in people with IDD. In their review of the literature on the use of non-invasive brain stimulation (NIBS) techniques, including tDCS, to modulate impulsivity in healthy subjects, Brevet-Aeby and colleagues [14] reported that tDCS can effectively modulate key facets of impulsivity including inhibitory control and delay discounting. A more recent review by Yang and colleagues [7] indicated that tDCS has a significant, albeit small, effect on modulating impulsivity in people with mental disorder. TDCS can be used to modulate a brain network involving the left dorsolateral prefrontal cortex (DLPFC) and limbic system (anterior insula, amygdala, anterior cingulate cortex). Disruption to this network, with reduced ‘top-down’ control from the DLPFC and an overactive limbic system, results in greater impulsivity [12]. Iwabuchi et al. [16] modulated this network using intermittent theta burst stimulation (iTBS) applied to left DLPFC, whereby iTBS significantly dampened fronto-insular effective connectivity. This mechanism might underpin the therapeutic effects of tDCS in reducing impulsivity.

### Objectives {7}

This study is the first randomised controlled clinical trial that aims to assess the efficacy of anodal tDCS in reducing RRI and incidents of aggression in people with IDD. We hypothesise that anodal tDCS applied to the left DLPFC will result in greater reductions in impulsivity and incidents of aggression than sham tDCS, demonstrating a clear treatment effect of tDCS in persons with IDD. Here, we outline the experimental protocol used to address our hypotheses.

### Trial design {8}

A single blind, parallel arms, randomised controlled trial design will be employed in this study. The trial is explanatory in nature and this type of trials aims to assess the efficacy of an intervention (i.e. tDCS) under controlled conditions that are well-defined [17].

## Methods: participants, interventions, and outcomes

### Study setting {9}

The study involves adults with mild IDD residing in the community, inpatient units, or care homes in the Southern Ontario Region of Canada.

### Eligibility criteria {10}

The inclusion criteria are as follows: (i) adults aged between 18 and 65 with mild IDD, (ii) a history of at least one incident of aggression in the last month, and (iii) consent to participate in the trial by the individual or their substitute decision-maker if they lack the capacity to consent to participate in research.

The exclusion criteria are as follows: (i) a history epilepsy, significant head injury or other neurological conditions, or brain surgery; (ii) having a metal in the brain or skull, a cardiac pacemaker, a central line, or a cochlear implant; (iii) current history of drug or alcohol misuse; and (iv) a history of adverse reaction to tDCS or having a sensitive scalp. Table 1 provides more information about the inclusion and exclusion criteria. A tDCS safety questionnaire will be administered to identify those who meet the exclusion criteria.

**Table 1:** Inclusion and exclusion criteria

Inclusion criteria	Exclusion criteria
Adults aged 18–65 years	History of epilepsy or seizures
Diagnosis of a mild intellectual developmental disability	History of acquired brain injury
History of 1 or more incidents of aggression in the last month	Having metal in the brain/skull, e.g. splinters, fragments, or clips
Consent to participate in the trial by the individual or their substitute decision-maker	Having a cochlear implant
	Having an implanted neuro-stimulator (e.g. direct brain stimulation, epidural/subdural stimulation, vagal nerve stimulation)
	History of brain surgery or procedure
	History of severe adverse reaction to tDCS
	Having a cardiac pacemaker or intracardiac lines
	Current alcohol or drug misuse
	Having a sensitive scalp

### Who will take informed consent? {26a}

In Canada, research ethics boards review applications in accordance with the principles set out in the “Tri-Council Policy Statement: Ethical Conduct for Research Involving Humans – TCPS 2 (2018)” [18]. TCPS defines decision-making capacity as “… the ability of prospective or actual participants to understand relevant information presented about a research project and to appreciate the potential consequences of their decision to participate or not participate.” (P.44) [18]. TCPS stipulates that for research involving individuals who lack capacity to consent, researchers should seek and maintain consent from an authorised third party, often referred to a substitute decision-maker (SDM), in accordance with the best interests of the individual concerned. In Ontario, the assignment of SDMs is governed either under the Substitute Decisions Act or the Health Care Consent Act based on either a treatment, personal, or financial decision and level of IDD does not preclude capacity to consent until formally queried and then an SDM is assigned. Hence, we would assume for the trial that potential participants will be deemed able to consent unless a SDM is already assigned or a Public Guardian Trustee is assigned due to no family members being accessible.

Members of the research team are acutely aware of the ethical issues regarding the enrolment of participants who lack capacity to consent to participate in research. The core ethical principles underpinning the TCPS policy on research will be adhered to, namely, respect for persons, concern for welfare, and justice [18].

The study will recruit participants [henceforth, the term ‘participant’ will be used throughout the protocol to refer to individuals with IDD undergoing the intervention] from intellectual developmental disability services in the Kingston area (or Southern Ontario Region). Posters will be placed publicly at Resource Centres in Kingston, and community living spaces where people with IDD reside. Participants will be identified by their psychiatrist, caregiver, or support worker. Capacity to consent to participate in research will be determined by the individual’s treating psychiatrist. Individuals designated to identify potential participants will ask prospective participants or their substitute decision-maker to complete a hospital “consent to be contacted for research” form. Study personnel (a research assistant or study coordinator) will then contact the individuals who have already consented to be contacted for research purposes either in person at the health service, by phone, or by email. If an individual is unable to provide consent, their substitute decision-makers will be approached. Study personnel will ensure that potential participants have not opted-out or withdrawn their consent or provided consent to be contacted for research purposes before initiating contact with participants or their substitute decision-makers.

Designated study personnel will review or read over the consent with the participant or their substitute decision-maker and answer any questions they have. Individuals must be able to understand that participation is completely voluntary and they can withdraw consent at any time and without giving a reason. To minimise coercion in the consenting process, the participant letter of information has been adapted to a grade 3 reading/comprehension level. The letter provides information about the side effects of tCDS using a simple language with visual aids. For example, it explains about tingling as follows: “A lot of people feel a tingling feeling under the pads. It should not hurt. This is felt when the electricity is turned on and just after. It is not scary.”

New information will be shared with participants and their substitute decision-makers at any time during their participation in the study as soon as it becomes available. Participants will be re-consented if necessary during the study at any study visit and the study will not proceed until the participant is re-consented. Participants or their substitute decision-makers can withdraw their consent at any time by expressing their desire to do so to study personnel.

### Additional consent provisions for collection and use of participant data and biological specimens {26b}

Not applicable.

## Interventions

### Explanation for the choice of comparators {6b}

The comparator is sham tDCS stimulation. The fundamental principle of a sham stimulation is that participants struggle to unblind themselves to the treatment. Ten seconds of stimulation is sufficient to keep individuals blinded to their treatment arm but is not enough stimulation to modulate brain activity. The validity of this sham protocol has been previously demonstrated [17].

### Intervention description {11a}

The Soterix tDCS kit [19] will deliver 20-min stimulation sessions using two 5×5 cm sponge electrodes. The stimulation montage will comprise left DLPFC anodal or sham stimulation. The anodal electrode will be placed over the area corresponding to the left DLPFC (F5 of the EEG10–20 international system) and the reference (cathodal) electrode over the right supraorbital ridge. The active stimulation condition will use a constant current of 2mA, delivered via current ramps over 10 s at the onset and offset of stimulation, respectively. For sham stimulation, the current will be delivered only in the first 10 s, after which the stimulation will cease but with the electrodes still in place throughout the session.

The intervention will be delivered in a private room at Providence Care Hospital in Kingston Ontario or at the residential home where the participant is residing if they are unable to travel. The intervention will be delivered by a research assistant under the supervision of the principal investigator.

### Criteria for discontinuing or modifying allocated interventions {11b}

Discontinuation rules for individual participants include withdrawal of consent, development of a coincidental health problem/illness, or exacerbation of aggressive behaviours expressed as a report of a serious violent incident and/or an increased incidence of self-harm or self-injurious behaviour as judged by their caregivers. Additionally, the study could stop early if the intervention significantly exacerbates aggressive, impulsive, or self-harming behaviours as judged by the trial investigators.

### Strategies to improve adherence to interventions {11c}

tDCS stimulation will be administered by a research assistant as per the study protocol. The research assistant will hold an undergraduate degree in health or social sciences. They will receive adequate training on the administration of the study protocol including tDCS. The principal investigator, who has expertise in tDCS, will provide the necessary training. To ensure adherence to the study protocol, two dummy runs will be conducted at the start of the trial, and these will involve the administration of the study questionnaires and tDCS. Additionally, the principal investigator will directly supervise the administration of the study protocol for the initial experiments that will involve participants.

### Relevant concomitant care permitted or prohibited during the trial {11d}

The participants will continue to receive usual care from mental health services or other support agencies for people with intellectual developmental disabilities. It is anticipated that the level of usual care will vary according to the need of the individual, and may include pharmacological and psychosocial interventions, some of which may target impulsivity and aggressive behaviours. It is envisaged that randomisation would counter balance the effects of these interventions across the study arms, such that additional effects could be attributed to tDCS.

### Provisions for post-trial care {30}

Patients will be informed what arm (treatment or sham) they were allocated to at the end of the study. They will also be advised to contact the study's principal investigator should they experience any adverse effects following each session. Additionally, participants will continue to receive usual care at the discretion of the treating psychiatrist or care team. It usually involves a combination of behavioural approaches, psychosocial interventions, and psychotropic medications.

### Outcomes {12}

#### Primary outcome measure

##### Aggression

The *Modified Overt Aggression Scale* (MOAS) [20] will be used as a repeated measure to assess aggressive behaviours at baseline, 1 week (day 10), and 1 month (day 38) following the last tDCS treatment. MOAS is an informant-rated scale which will be completed by a member of care staff who have professional knowledge of the participant. MOAS has four domains (Verbal Aggression, Aggression Against Property, Autoaggression, and Physical Aggression) which are weighted and each rated on a 5-point scale (0–4). Scoring of MOAS yields weighted scores, total and by domain. MOAS is considered to be a reliable measure of aggressive behaviours in people with IDD [21].

#### Secondary outcome measures

##### Behavioural impulsivity

The *Stop Signal Task (SST)* (Psytoolkit.org) is a behavioural measure of RRI. It is a repeated measure that will be completed by each participant at baseline and following the third tDCS treatment (day 3) with Stop Signal Reaction Time (SSRT) as the outcome of interest. SSRT is a continuous measure. It is defined as the mean reaction time on go trials minus the mean Stop Signal Delay at which the participant successfully withholds a response on 50% of the trials. Lower SSRT values corresponds to higher impulsivity. SST is a measure of inhibitory control of which RRI is an example. Measures of inhibitory control have been validated for use in people with IDD [22].

##### Maladaptive behaviours

*The Behavior Problems Inventory Short Form* (BPI-S) [23] is an informant-rated instrument which is used to assess maladaptive behaviours in people with IDD. Changes in maladaptive behaviours will be measured at baseline, 1 week (day 10), and 1 month (day 38) following the last tDCS treatment. BPI-S is a 30-item scale that assesses behaviour problems on three domains: self-injurious, aggressive/destructive, and stereotyped behaviours. It is a repeat measure in which scoring yields total and domains scores for frequency for all domains as well as severity scores for self-injurious and stereotyped behaviours domains. It measures frequency on a 5-point scale (0=never/no problem, 1=monthly, 2=weekly, 1=daily, 4=hourly). It measures severity on a 3-point scale (1=mild, 2=moderate, 3=severe). BPI has been developed for use in people with IDD with good psychometric properties [24].

##### tDCS adverse effects

Participants will be monitored for side effects of Active/Sham tDCS treatment using a tDCS Adverse Effects Questionnaire adapted from Brunoni et al. [25]. Following each treatment session, participants will be asked to record any side effects related to tDCS. Each item is measured on a 4-point scale (0=absent; 1=mild, 2=moderate, 3=severe).

##### Treatment Acceptability Questionnaire

The acceptability of tDCS as a healthcare intervention will be assessed using a Treatment Acceptability Questionnaire developed for the purpose of this study. The questionnaire draws from the work of Sekhon et al. [26] who outlined acceptability measurement methods for different stages of healthcare research based on a theoretical framework of acceptability. The questionnaire has 7 items. Each item is measured on a 3-point scale (1=not satisfied at all, 2=neutral, 3=very satisfied) with corresponding emojis. Participants will be asked to complete the questionnaire after the last tDCS session.

#### Additional measures

##### Trait impulsivity

To account for the effects of trait impulsivity on the outcome measures, the *Barratt Impulsiveness Scale version 11 (BIS-11)* [27] will be completed by each participant once at baseline, using the version adapted for use in people with IDD [28, 29]. This is a 30-item scale and each item is measured on a 4-point Likert scale (1=almost never, 2=sometimes, 3=often/a lot, 4= almost always/always). The first two points are denoted with dotted bars and the last two with solid bars for ease of reference. BSI-11 scoring yields a total score out of 120, and subscores for three higher order factors (attentional, motor and non-planning).

### Participant timeline {13}

**Fig. 1:**
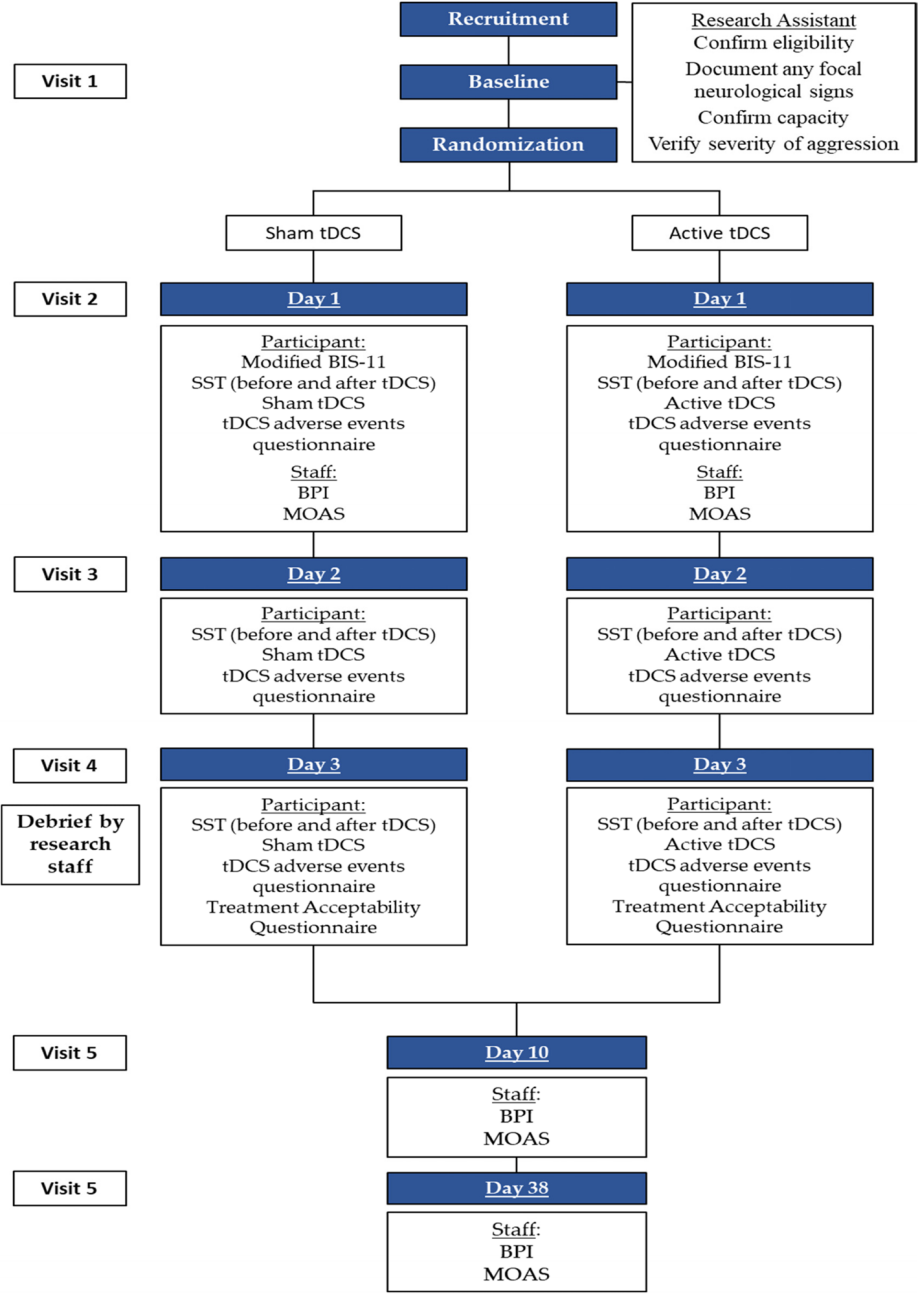
Trial CONSORT diagram. Stop signal task (SST), Behavior Problems Inventory (BPI), Barratt Impulsiveness Scale version 11 (BIS-11), Modified Overt Aggression Scale (MOAS)

Figure 1 outlines the proposed study procedure. Eligible participants will have a baseline clinical visit to confirm eligibility criteria and patient capacity, verify severity of aggressive behaviours, and obtain informed consent from participant or designated substitute decision-maker. Once enrolled, participants will be randomised into one of two study arms using a 1:1 allocation ratio. Participant blinding will be maintained but the research team will be unblinded to study arm.

Participant healthcare records will be examined to obtain information concerning the diagnosis of IDD, demographics, number and duration of admissions to psychiatric institutions, and current psychotropic medication regime. A member of staff at the participant’s residence with good professional knowledge of the participant will be asked to complete the BPI and MOAS at baseline and 1 week after the last tDCS stimulation. At baseline, participants will be asked to complete the modified BIS-11, and SST. Three tDCS sessions will be delivered over three successive days (one session daily). Participants will be asked to repeat SST at the end of the third session (Fig. 1). Additionally, a tDCS adverse effects questionnaire will be administered following each tDCS treatment to detect adverse effects. At 1 week and also 1 month following the third and final treatment, participants will complete the BPI and MOAS and will be debriefed to unblind them to treatment arm.

### Sample size {14}

A power calculation conducted using repeated measures ANOVA (G*Power) yielded a total sample size of 50 participants (effect size=0.30, power=95%, *α* = 0.05). To allow for an attrition rate of 20%, we will aim to recruit 60 participants (30 per arm). These parameters were based on other studies involving the use of tDCS to modulate cognitive and behavioural functioning [30, 31]. Indeed, Minarik and colleagues [30] recommended small to intermediate effect sizes (between *d*=0.4 and *d*=0.5) for tDCS studies. We opted for an effect size of *d*=0.3 to improve precision. Furthermore, sample size calculation by Cosmo and colleagues [31] yielded 25 participants per arm for a power of 80% and bidirectional type I error probability of 0.05%. To allow for an attrition rate of 20%, a final sample size of 60 was considered sufficient by Cosmo and colleagues to detect a 50% difference in performance on the inhibitory control task in the active tDCS group, and a 10% difference in the sham tDCS group. While we acknowledge the importance of accounting for clustering by in-patient/care home, given our clinical experience, we anticipate that most of the participants will be recruited from the community. Furthermore, given the special nature of the population in this study and the funding constraints, it would be unrealistic to aim to recruit larger numbers. Nevertheless, we believe that the sample size of 30 participants per study arm is sufficiently large to detect differences between the effects of active and sham tDCS.

### Recruitment {15}

Participants will be recruited over 24 months from clinics, inpatient units, and community residential homes for people with IDD.

## Assignment of interventions: allocation

### Sequence generation {16a}

Participants will be randomly assigned to receive either active or sham tDCS using block randomisation to ensure that an equal number of participants is assigned to each study arm. The research assistant or trial coordinator will be initially blind to the allocation sequence.

### Concealment mechanism {16b}

The allocation sequence will be communicated to the research assistant or trial coordinator after the completion of baseline measures including BPI-S, BIS-11 and MOAS.

### Implementation {16c}

A member of the research team other than the principal investigator will generate the allocation sequence. The study’s research assistant or coordinator will enrol participants, complete the outcome measures, and administer the interventions.

## Assignment of interventions: blinding

### Who will be blinded {17a}

This a single-blinded study where participants will be blinded to treatment allocation. Professionals who complete MOAS and BPI-S will also be blinded to treatment allocation. To minimise bias, the principal investigator and research assistant will be initially blinded to the allocation sequence. This will be generated by a Queen’s University independent faculty member who is not involved in the study, and then communicated to the research assistant administering the intervention after the completion of baseline measures. It is anticipated that having the research assistant unblinded is unlikely to introduce assessment bias since all the study questionnaires will be completed by the participants and professionals involved in their care, who will be blinded to the treatment allocation. Participants will only be unblinded to the treatment allocation if they developed a serious adverse reaction such as self-harm or serious violence. To assess the robustness of blinding, participants will be asked to guess their treatment allocation after the last tDCS session.

### Procedure for unblinding if needed {17b}

As the study is single-blinded, the treatment administrator will always know what treatment arm the participants are in, so that in the event of an emergency, administrators can immediately unblind the participant and/or emergency personnel.

## Data collection and management

### Plans for assessment and collection of outcomes {18a}

This information is provided under “Participant timeline” section of the protocol.

### Plans to promote participant retention and complete follow-up {18b}

The research team will work closely with participants and their caregivers and substitute decision-makers, where applicable, to promote retention and complete follow-up. A travel allowance is available to those who are unable to travel to the study administration site.

### Data management {19}

Access to electronic and paper data will be limited to the research team. The data will also be accessible to the research governance team at Providence Care Hospital and Queen’s University to ensure the quality of research conduct. The electronic data will be encrypted and stored in a password-protected computer. A backup copy will be stored in a password-protected storage device. Paper files will be stored in a locked filing cabinet at Providence Care Hospital. A master file linking identify codes with participant identifiers, accessible only by the principal investigator and research coordinator, will be stored separately from the study data.

### Confidentiality {27}

Access to the participants’ medical records and study data will be limited to authorised research personnel. Access to electronic data will be password protected and auditable. Electronic data will be stored on a hospital or other institutional network with firewalls and other security and back-up measures in place. Data stored on laptops or mobile devices will be encrypted. Paper copies of study data will be stored in locked filing cabinets in a secure location at Providence Care Hospital in Kingston Ontario. A master linking logs with identifiers, accessible only by the principal investigator and research coordinator, will be stored separately from the study data.

### Plans for collection, laboratory evaluation, and storage of biological specimens for genetic or molecular analysis in this trial/future use {33}

Not applicable.

## Statistical methods

### Statistical methods for primary and secondary outcomes {20a}

Behavioural outcome measures will be analysed using the Statistical Package for Social Sciences (SPSS; Version 26). A 2 × 2 Repeated Measures ANOVA will be performed with group allocation (active tDCS vs. sham tDCS) and time (pre- vs. post-tDCS stimulation). Post tDCS measures for MOAS, SST, and BPI-S will be treated separately as repeated measures. Total scores on BIS-11 will be entered into the analysis as a covariate. Significant main effects and interactions will be explored using simple effects analysis. For instance, within-subject factors will be used to compare changes in outcome variables between the groups over time. A greater reduction in the total scores on MOAS in the active tDCS group than the sham tDCS group would be considered a successful primary outcome. Greater reductions in the total scores on BPI and greater increases in Stop Signal Reaction Time in the active tDCS group than the sham tDCS group would be considered successful secondary outcome. A *p* value of < 0.05 will be considered as statistically significant for the primary outcome measure. For secondary outcomes, multiple comparisons will be controlled for with Bonferroni correction and a *p* value of less than 0.0125 will be considered statistically significant.

### Interim analyses {21b}

Interim analysis will be conducted once half (30) of the participants have been recruited. Study team members will perform this interim analysis. Stopping rules include: withdrawal of consent, development of a coincidental health problem/illness, exacerbation of aggressive behaviours expressed as a report of a serious violent incident and/or an increased incidence of self-harm or self-injurious behaviour. The principal investigator and the Research Ethics Board will make the final decision about early trial termination.

### Methods for additional analyses (e.g. subgroup analyses) {20b}

Subgroup analyses will be conducted by psychiatric diagnosis (psychosis vs. no psychosis). The latter is important since psychosis is a key risk factor for aggression.

### Methods in analysis to handle protocol non-adherence and any statistical methods to handle missing data {20c}

Data will be analysed on an intention-to-treat basis such that the last observations will be carried forward. Otherwise, depending on the pattern of missing data in the last observation, e.g. if missing at random, multiple imputations technique will be used to deal with missing data [32].

### Plans to give access to the full protocol, participant-level data, and statistical code {31c}

The full protocol will be made publicly available on Clinicaltrials.gov. Anonymised participant-level data and statistical code can be provided by the principal investigator upon request.

## Oversight and monitoring

### Composition of the coordinating centre and trial steering committee {5d}

A research management group comprising the trial investigators will be established to manage the overall governance of the project and day-to-day operations. The group will meet monthly to discuss progress and to ensure that the study is conducted in accordance with the requirements of the research ethics approval. While it is acknowledged that a trial steering committee can play an important role in maintaining the quality of study conduct, financial constraints precluded the appointment of such committee. However, this study will be conducted under the auspices of Queen’s University and Providence Care Hospital, where rigorous research governance structures are in operation to ensure the quality of study conduct.

### Composition of the data monitoring committee, its role, and reporting structure {21a}

The research management group will be responsible for data monitoring. While we acknowledged that data monitoring is best conducted by an independent committee, financial constraints precluded the appointment of such committee. However, as mentioned earlier, robust research governance procedures are in operation at Queen’s University and Providence Care Hospital to ensure the quality of study conduct.

### Adverse event reporting and harms {22}

tDCS is considered to be a safe technique, but some small risks are recognised. A review of 567 tDCS sessions and from questionnaire responses from 102 participants [33] reported that the most common side-effect (reported by 70% of participants) is that of a tingling sensation under the electrodes. This is present during and shortly after the period of stimulation and has no adverse effects or risks. Fatigue or tiredness during the stimulation is the next common report (by about 35% of participants), and this may continue for a short period afterwards. This may occur when prolonged and uninteresting tasks are used during the experiment. Headaches after stimulation may occur in less than 10% of the participants. Headaches are usually mild and can be treated with normal over-the-counter painkillers, if required. There is no evidence that tDCS leads to any change in frequency or severity of headaches. Overall, less than 20% of the participants rated the stimulation procedure as mildly unpleasant and 80% reported that it was not unpleasant. In theory, tDCS might induce seizures, but this has never been reported in the scientific literature.

Potential risks will be completely disclosed to the participants as well as what to do should participants experience any side effects. For instance, should the tingling sensation become painful, the treatment will be stopped. The psychological or emotional risk is minimal; this risk will be mitigated with education and assurance from the study team. Additionally, participants will be debriefed at the end of each session and advised to contact the principal investigator if they experienced any adverse effects as a result of participation in the study. If a serious adverse event occurs during the treatment sessions, local staff (clinic, hospital, institutional) will be immediately notified and emergency protocols followed. We are not anticipating any adverse events serious or otherwise. However, should one occur, it will be documented and reported to the Research Ethics Board.

### Frequency and plans for auditing trial conduct {23}

On-site study monitoring will be conducted by designated Providence Care Hospital clinical coordinators who will monitor study safety as serious adverse effects are reported or at least every 6 months.

### Plans for communicating important protocol amendments to relevant parties (e.g. trial participants, ethical committees) {25}

Approval to implement important protocol modifications (e.g. changes to eligibility criteria, outcomes, analyses) will be sought from the Queen’s University Health Sciences Research Ethics Board (HSREB). Upon receiving approval, the changes will be communicated in writing to relevant parties (e.g. investigators, trial participants) and the trial record on ClinicalTrials.gov will be amended accordingly.

### Dissemination plans {31a}

The study trial results will be communicated to healthcare professionals, academics and other relevant groups (e.g., policy makers) via conference presentations and publication in a peer-reviewed journal. The results will be communicated to participants via a poster using a simple language and visual aids. Approval to distribute the poster will be sought at a later stage from the research ethics board.

## Discussion

This will be the first randomised controlled trial in the world to assess the efficacy of anodal versus sham tDCS applied to the left DLPFC to reduce impulsivity and aggression in adults with IDD. The proposed study will help elucidate the role of tDCS in reducing impulsivity and incidents of aggression in this population. Assessing the efficacy of repeated tDCS, for instance, delivered daily over a few weeks, and the parameters required to achieve optimal effects will be important considerations in future studies. The results of this study will prompt further research in the field, paving the way for developing targeted interventions for modulating impulsivity and aggressive behaviours in people with developmental disabilities.

## Trial status

Protocol version 5 dated January 7, 2022

Date recruitment begins: July 01, 2021

Approximate date when recruitment will be completed: June 30, 2023.

## Data Availability

The study investigators will have access to the final trial dataset, and there is no contractual agreements that limit such access for investigators. Authorised individuals from Queen’s University and Providence Care Hospital will also have access to the final trial to ensure that the study is conducted in accordance with the parameters of ethics approval.

## Abbreviations

BIS-11: Barratt Impulsiveness Scale version 11
BPI-S: Behavior Problems Inventory Short Form
DLPFC: Dorsolateral prefrontal cortex
IDD: Intellectual developmental disabilities
iTBS: Intermittent theta burst stimulation
MOAS: Modified Overt Aggression Scale
RRI: Rapid-response impulsivity
SSRT: Stop Signal Reaction Time
SST: Stop Signal Task
tDCS: Transcranial direct current stimulation
